# Thrombotic and Infectious Morbidity Are Associated with Transfusion in Posterior Spine Fusion

**DOI:** 10.1007/s11420-017-9545-9

**Published:** 2017-02-14

**Authors:** Daniel J. Johnson, Christine C. Johnson, David B. Cohen, Joshua A. Wetzler, Khaled M. Kebaish, Steven M. Frank

**Affiliations:** 1 Vanderbilt University School of Medicine, Nashville, TN 37232 USA; 2 Hospital for Special Surgery, New York, NY 10021 USA; 3 The Johns Hopkins Medical Institutions, Johns Hopkins Hospital, 1800 Orleans Street, Sheik Zayed Tower 6208, Baltimore, MD 21287 USA

**Keywords:** transfusion, outcomes, posterior spinal fusion, infection, deep venous thrombosis

## Abstract

**Background:**

Although previous investigators have established an association between blood transfusion and adverse outcomes, the relative frequency of different morbid events and the association with transfusion dose are not well understood.

**Questions/Purposes:**

The purpose of the study is to characterize the relationship between blood transfusion and different types of morbidity after posterior spine fusion.

**Methods:**

We retrospectively analyzed electronic medical records for 963 patients who underwent posterior spinal fusion surgery at a single institution, of which 603 (62.6%) received an allogeneic blood transfusion. Then, we assessed patient and surgical characteristics in a risk-adjusted fashion to identify various morbid event rates and independent predictors in these adverse outcomes.

**Results:**

Compared to the non-transfused patients, transfused patients had a higher incidence of any morbid event (9.1 vs. 2.5%. *P* < 0.0001), thrombotic events (4.6 vs. 1.1%, *P* = 0.0025), and hospital-acquired infections (2.3 vs. 0.6%, *P* = 0.039). Renal, respiratory, and ischemic morbidity occurred less frequently and were not more common in transfused patients. Risk-adjusted analysis revealed a dose-response effect, whereby for each unit of allogeneic blood transfused, the risks of any morbid event (OR 1.183; 95% CI 1.103–1.274; *P* < 0.0001), thrombotic complication (OR 1.104; 95% CI 1.032–1.194; *P* = 0.0035), and infectious complication (OR 1.182; 95% CI 1.077–1.332; *P* = 0.0002) were increased.

**Conclusion:**

Our data demonstrate risk-adjusted and transfusion dose-related increases in perioperative morbidity, with thrombotic and infectious events being the most common.

**Electronic supplementary material:**

The online version of this article (doi:10.1007/s11420-017-9545-9) contains supplementary material, which is available to authorized users.

## Introduction

Spinal fusion is among the top 10 surgical procedures associated with blood transfusion [27], and the utilization of blood for this procedure is increasing [33]. Despite its widespread use, blood transfusion is not without risks [1]. Perioperative blood transfusion has been associated with hospital-acquired infection, [1, 16, 24, 25, 28, 32], and this finding was recently demonstrated in a meta-analysis of randomized trials [24], where a liberal transfusion strategy was a risk for infection, especially in orthopedic surgery patients.

Although the relationship between transfusion and infection after spine surgery has been established, the association of transfusion with other complications is unclear [16, 25, 32]. For example, in a retrospective analysis of 300 patients undergoing spine surgery with substantial blood loss, blood transfusion was associated only with surgical site infection, and not with other complications; however, it is important to note that the authors included very few noninfectious complications in their analysis [22]. In contrast, a large database analysis revealed that even low-dose blood transfusion (even 1 unit) conferred an increased risk of any major or minor complication after elective spine surgery [28]. However, the authors did not provide data on the incidence of individual complications, and “major” complications varied widely by definition, from postoperative wound breakdown to coma. Larger sample sizes with detailed perioperative data are necessary to assess the relationship between transfusion and noninfectious complications. Additionally, most previous studies have used broad primary outcomes, such as “major complications” or “any perioperative complication,” which precludes any analysis of the relationship between transfusion and specific morbid event rates.

A better understanding of the risk-benefit balance between anemia, transfusion, and clinical outcomes after spinal fusion is necessary to optimize clinical outcomes and reduce healthcare costs [10]. Such a risk-benefit analysis is especially important because transfusion is the most common procedure performed in US hospitals [12], and the Joint Commission has determined that transfusion is one of the top five overused procedures [18]. The purpose of this study was to determine (1) if perioperative red blood cell (RBC) transfusion is associated with postoperative morbidity (including noninfectious complications) and (2) if a dose-response relationship exists between transfusion and the likelihood of developing these complications.

## Patients and Methods

After receiving approval from the institutional review board at our institution, we acquired electronic medical record data from a web-based intelligence portal (IMPACT Online, Haemonetics Corp., Braintree, MA), our hospital billing database (Datamart), and the anesthesia information management system (Metavision; iMdSoft, Dedham, MA) for patients who were discharged from our institution between January 2009 and January 2015. Current Procedural Terminology (CPT) codes were queried to identify consecutive patients who underwent posterior spinal fusion surgery. CPT codes included were 22612, 22630, 22633, 22800, 22802, and 22804 (Table 1). Only patients over the age of 18 were included in the analysis.

**Table 1:** Description of CPT codes

CPT code	CPT code description
22612	Posterolateral fusion, lumbar (first segment)
22630	Posterior interbody fusion, lumbar
22633	Combined fusion, posterolateral fusion, with posterior interbody fusion
22800	Arthrodesis, posterior, for spinal deformity; less than or equal to 6 vertebral segments
22802	Arthrodesis, posterior, for spinal deformity; 7 to 12 vertebral segments
22804	Arthrodesis, posterior, for spinal deformity; 13 or more vertebral segments

Our use of these databases and our quality control methods have been described previously [8, 9]. Collected data included up to 29 pre-hospital comorbidities for each patient that were used to determine the Charlson Comorbidity Index [4], the number of vertebral levels surgically fused, the number of allogeneic RBC units each patient received throughout the hospitalization, amount of autologous blood transfused intraoperative, length of stay, estimated blood loss, nadir hemoglobin during hospitalization, and duration of surgery. Vertebral levels were dichotomized into two groups: less than or equal to three levels and greater than three levels.

In-hospital morbid events were determined by ICD-9 codes upon discharge, as we have described previously [26, 30]. Outcomes of interest included transient ischemic attack (TIA), cerebrovascular attack (CVA), myocardial infarction (MI), ventilator-associated pneumonia, kidney injury, postoperative infection, sepsis, drug-resistant infection, *Clostridium difficile* infection, deep venous thrombosis (DVT), pulmonary embolism (PE), and disseminated intravascular coagulation (DIC). Postoperative infection includes a postoperative urinary tract infection, a surgical site infection, pneumonia, or other infectious complications occurring in the postoperative period. A thrombotic event was defined as one or more of the following: DIC, DVT, or PE. A hospital-acquired infection was defined as one or more of the following: postoperative infection, sepsis, drug-resistant infection, or *C. difficile* infection. An ischemic event was defined as one or more of the following: TIA, CVA, or MI. These groupings were not mutually exclusive, for instance, a patient could have both a TIA and an MI but that would only count as one ischemic event.

Chi-squared and Fisher’s exact tests were used to compare dichotomous variables, and Student’s *t* test was used to analyze continuous variables. The Wilcoxon rank sum test was used to analyze data that were not normally distributed. Normality was tested with the Shapiro-Wilk test. Risk for transfusion and perioperative morbidity were analyzed in both an unadjusted and risk-adjusted fashion by univariable and multivariable logistic regressions, respectively. Variables entered into the logistic regression model include those clinical factors that have been shown in previous studies to be associated with adverse outcomes, and those factors that were associated with morbidity upon univariate testing in the current study. Estimated blood loss was entered as quartiles since as a continuous variable the interaction between RBCs transfused and estimated blood loss rendered the model unstable. Continuous variables that were normally distributed are presented as mean ± SD, whereas other variables are presented as median (interquartile range). *P* < 0.05 (by two-tailed tests) was used to define significance. Non-adjusted and risk-adjusted odds ratios (ORs) and 95% confidence intervals (CIs) are reported. Analyses were generated with JMP version 12 (SAS Institute, Inc., Cary, NC).

## Results

One thousand five hundred sixty-three patients had a CPT code which indicated a procedure that included a type of spinal fusion (Table 1). Six hundred patients were excluded for age <18 years. Nine hundred sixty-three patients were included in our analysis. The cohort had slightly more women (*N* = 553, 57.4%) than men, and the average age was 56 ± 16 years (Table 2). The median estimated surgical blood loss was 800 mL (400, 1,400). Only 4.6% of our cohort received tranexamic acid, as this medication was only recently approved for select cases at our institution. The transfused group of patients had longer surgeries and more blood loss.

**Table 2:** Patient and surgical characteristics by transfusion status

Characteristic	All patients (n = 963)	Transfused^a^ (n = 603)	Not transfused (n = 360)	P value^b^
Age, mean ± SD	56 ± 16	59 ± 15	52 ± 16	<0.0001
Male (%)	410 (42.6)	215 (35.7)	194 (54.1)	<0.0001
Race (%)				0.07
Caucasian	737 (76.5)	456 (75.6)	281 (78.1)	
African American	131 (13.6)	93 (15.4)	38 (10.6)	
Other	95 (9.9)	54 (9)	41 (11.4)	
Tranexamic acid use (%)	44 (4.6)	27 (4.5)	17 (4.7)	0.87
Charlson score (median, IQR)	1 (0, 2)	1 (0, 2)	0 (0, 1)	<0.0001
BMI	28.4 (24.5, 32.7)	28.4 (24.4, 33.0)	28.5 (24.7, 32.3)	0.43
Nadir hemoglobin	8.9 ± 1.4	8.2 ± 0.9	10 ± 1.3	<0.0001
>3 vertebral level fusions (%)	387 (40.2)	303 (50.3)	84 (23.3)	<0.0001
Estimated blood loss (mL)	800 (400, 1,400)	1,100 (700, 1,700)	400 (300, 700)	<0.0001
Surgical time (hr)	5.7 (4.4, 7.2)	6.5 (4.9, 8)	4.8 (3.9, 4.9)	<0.0001
Autologous blood (mL)	109 ± 241	165 ± 287	17 ± 74	<0.0001

BMI body mass index, IQR interquartile range, SD standard deviation, mL milliliters, min minutes

^a^Transfused allogeneic blood

^b^Difference between transfused and not transfused groups

A total of 603 patients (62.6%) received a blood transfusion, with 419 (43.5%) receiving blood intraoperatively, 499 (51.8%) receiving blood postoperatively, and 311 (32.3%) receiving blood during both time periods. Of the 963 patients included in the analysis, 505 (52.4%) were on the orthopedic surgery service and 458 (47.6%) were on the neurosurgery service. In the univariate analysis, significant predictors of transfusion were increased age (*P* < 0.0001), increased Charlson score (*P* < 0.0001), female sex (*P* < 0.0001), increased estimated blood loss (*P* < 0.0001), and increased surgical time (*P* < 0.0001) (Table 2).

Compared with the non-transfused patients, transfused patients had a higher incidence of any morbid event (9.1 vs. 2.5%. *P* < 0.0001), thrombotic events (4.6 vs. 1.1%, *P* = 0.0025), and hospital-acquired infections (2.3 vs. 0.6%, *P* = 0.039) (Table 3). Renal, respiratory, and ischemic morbidity occurred less frequently and had similar rates in transfused and non-transfused patients (renal morbidity 1.5 vs. 1.1%; *P* = 0.78; respiratory morbidity: 0.7 vs. 0%; *P* = 0.3; ischemic morbidity: 0.7 vs. 0%; *P* = 0.3).

**Table 3:** Outcomes after transfusion

Outcome	Transfused (n = 603)	Not transfused (n = 360)	P value
Any morbid event, n (%)	55 (9.1)	9 (2.5)	<0.0001
Infection, n (%)	14 (2.3)	2 (0.6)	0.04
Sepsis	2 (0.3)	0 (0)	0.53
SSI	10 (1.7)	2 (0.6)	0.23
Drug-resistant infection	1 (0.2)	0 (0)	0.99
Clostridium difficile	1 (0.2)	0 (0)	0.99
Thrombotic complication, n (%)	28 (4.6)	4 (1.1)	0.003
Deep venous thrombosis^a^	13 (2.2)	1 (0.3)	0.02
Pulmonary embolism^a^	15 (2.5)	2 (0.5)	0.04
DIC	6 (1)	1 (0.3)	0.27
Renal complication, n (%)	9 (1.5)	4 (1.1)	0.78
Respiratory complication n (%)	4 (0.7)	0 (0)	0.3
Ischemic event, n (%)	4 (0.7)	0 (0)	0.3
Death, n (%)	1 (0.2)	0 (0)	0.99

SSI surgical site infection, DIC disseminated intravascular coagulation

^a^Six patients had a deep venous thrombosis and pulmonary embolism

The association between allogeneic blood transfusion and perioperative morbidity (any morbid event) was assessed by univariable and multivariable analyses (Table 4). Univariable analysis showed that increasing Charlson score, vertebral levels fused, estimated blood loss, surgical time, and number of RBC units were associated with an increased risk of occurrence of any perioperative morbid event. In the multivariable analysis, after controlling for age, sex, race, Charlson score, BMI, number of vertebral levels, estimated blood loss, and surgical duration, increased allogeneic RBC transfusion (OR 1.183 per unit; 95% CI 1.103–1.274), and increased Charlson score (OR 1.166 per point; 95% CI 1.004–1.339) were associated with an increased risk of perioperative morbidity (the occurrence of any morbid event).

**Table 4:** Risk factors for perioperative morbidity^a^

	Univariable		Multivariable^b^	
	OR (95% CI)	P value	OR (95% CI)	P value
Age	1.009 (0.986–1.034)	0.3	0.999 (0.981–1.019)	0.95
Male sex	1.053 (0.503–2.162)	0.84	0.886 (0.485–1.596)	0.69
Patient race				
Caucasian	1.00 (reference)		1.00 (reference)	
African American	0.955 (0.274–2.581)	0.91	1.211 (0.5, 2.63)	0.65
Other	1.536 (0.469–4.07)	0.28	2.11 (0.813–4.922)	0.12
Charlson score	1.299 (1.088–1.533)	<0.0001	1.166 (1.004–1.339)	0.045
Body mass index	1.003 (0.943–1.065)	0.88	1 (0.956–1.045)	0.99
Number of levels (>3 vs ≤3)	2.598 (0.128, 5.815)	0.003	1.511 (0.807–2.852)	0.2
Estimated blood loss^c^	1.533 (1.195, 1.989)	0.0007	0.937 (0.65–1.247)	0.72
Surgical duration (hours)	1.224 (1.102, 1.357)	0.0002	1.026 (0.878–1.193)	0.74
RBC units^d^	1.188 (1.094–1.293)	<0.0001	1.183 (1.103–1.274)	<0.0001

RBC red blood cell

^a^Perioperative morbidity includes having at least one of the following morbid events: transient ischemic attack, cerebrovascular attack, myocardial infarction, ventilator-associated pneumonia, kidney injury, surgical site infection, sepsis, drug-resistant infection, Clostridium difficile infection, deep venous thrombosis, pulmonary embolism, and disseminated intravascular coagulation

^b^Independent variables in the model included age, sex, race, Charlson score, body mass index, surgical service, number of levels, estimated blood loss (as quartiles), and surgical duration

^c^Modeled in quartiles—(1) <400 mL, (2) 400–800 mL, (3) 800–1400 mL, and (4) >1400 mL

^d^RBC units as a continuous variable (Odds ratio is effect per unit transfused)

**Fig. 1:**
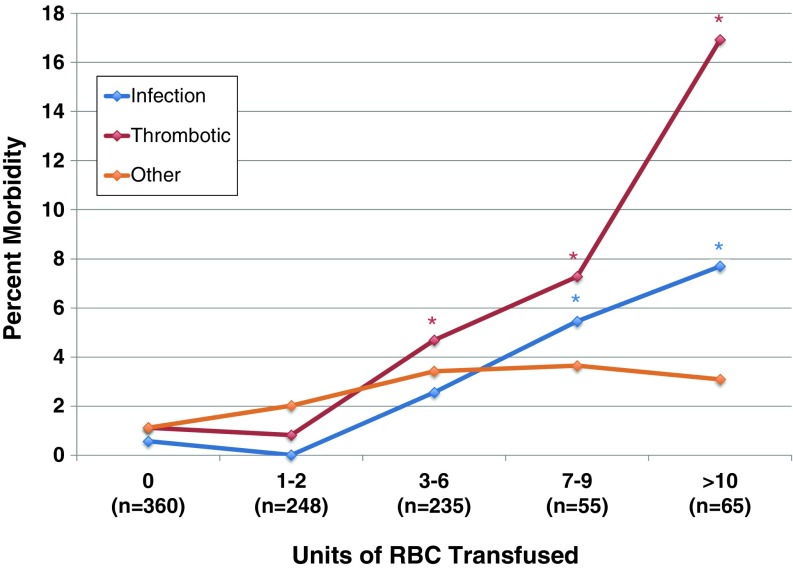
Transfusion dose-response relationship for specific morbid events, including thrombotic, infectious, and other (ischemic, renal, or respiratory) complications. *P < 0.05 in comparison to patients who received 0 units. *RBC* red blood cells.

**Fig. 2:**
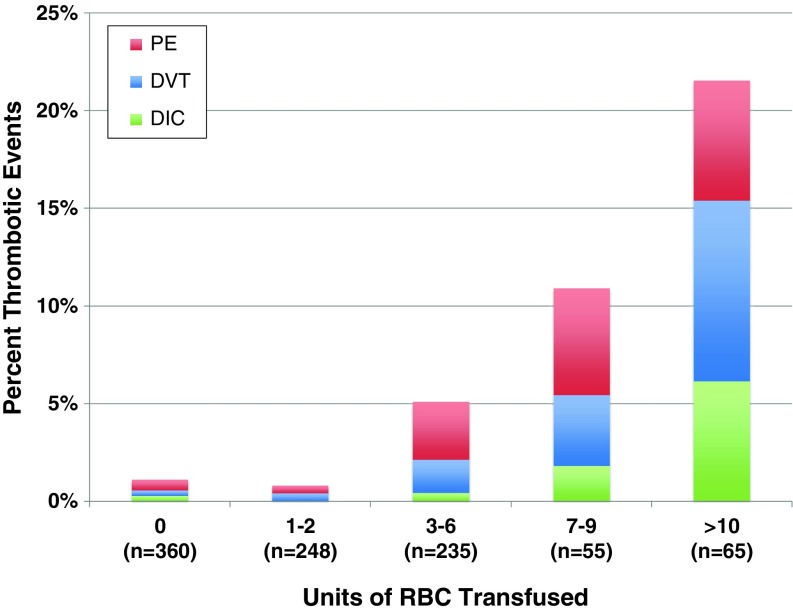
Transfusion dose-response relationship across multiple transfusion doses for specific thrombotic morbidities, including pulmonary embolism (*PE*), deep venous thrombosis (*DVT*), and disseminated intravascular coagulation (*DIC*). *RBC* red blood cells.

**Fig. 3:**
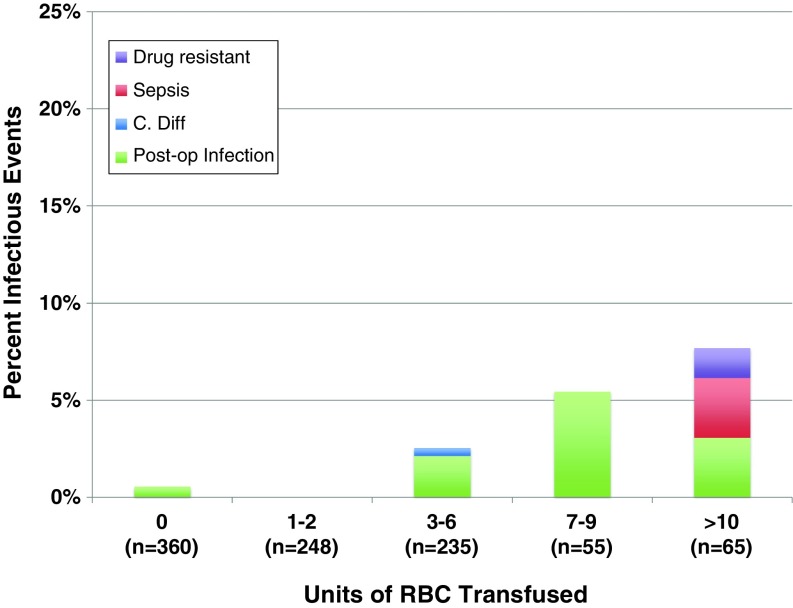
Transfusion dose-response relationship across multiple transfusion doses for specific infectious morbidities, including drug-resistant infection, sepsis, *Clostridium difficile*, and postoperative infection. *RBC* red blood cells.

**Fig. 4:**
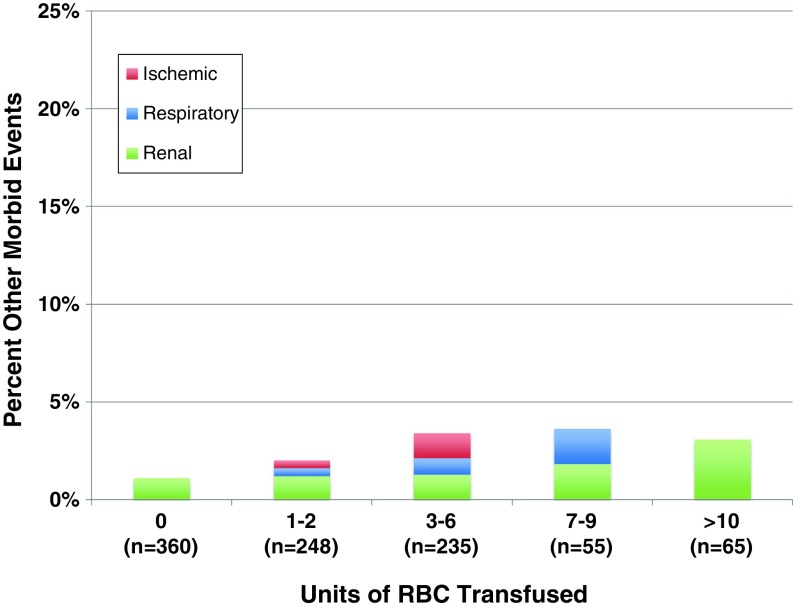
Transfusion dose-response relationship across multiple transfusion doses for ischemic (stroke, transient ischemic attack, or myocardial infarction), respiratory, and renal complications. *RBC* red blood cells.

The relative occurrence rates of the different morbid events are shown according to the transfusion dose in Fig. 1. It is notable that a dose of 1–2 RBC units was not associated with a change in morbid event rates, but a dose of ≥3 RBC units was a threshold at which morbidity increased significantly. Overall, thrombotic complications occurred in 3.3% of all patients. These thrombotic complications were relatively evenly distributed among DIC, DVT, and PE, as all three types of thrombotic event increased with increasing transfusion dose (Fig. 2). Overall, infectious complications occurred in 1.7% of all patients and increased in frequency with increasing transfusion dose (Figs. 1 and 3). These infections included 12 surgical site infections, two cases of sepsis, one drug-resistant infection, and one case of *C. difficile*. Renal complications occurred with an overall frequency of 1.4%, and ischemic and respiratory morbid events occurred with an overall frequency of less than 1%. Ischemic, respiratory, and renal morbid events, despite being less likely, tended to increase with increasing dose of RBC transfused, demonstrating a possible dose-response relationship (Fig. 4)

In a risk-adjusted multivariable analysis controlling for age, sex, race, Charlson score, body mass index, levels fused, intraoperative blood loss, and surgical duration, RBC transfusion dose (per unit) was independently associated with an increased odds of any infection (OR 1.182; 95% CI 1.077–1.332; *P* = 0.0002), and increased odds of a thrombotic event (OR 1.104; 95% CI 1.032–1.194; *P* = 0.0035).

## Discussion

Our findings demonstrate that allogeneic RBC transfusion is associated with a dose-dependent increased risk for infectious and thrombotic complications spinal fusion, even after risk-adjustment for other known predictors of perioperative morbidity. As the rate of spinal fusion surgery increases in the USA, particularly among older patients [21], understanding the relationship between transfusion and clinical outcomes is relevant to optimizing clinical care while reducing costs [10]. Although the association between transfusion and infection after spine procedures has been described previously [16, 25, 32], to our knowledge, this study is the first to investigate noninfectious outcomes and the relationship between transfusion and these complications.

Potential limitations in our study include the retrospective observational design, which is only able to suggest association not causation. To increase the validity of our findings we controlled for potential confounders, such as intraoperative blood loss, number of spinal fusion levels, operative time, and comorbid conditions. After such risk-adjustment, transfusion remained a dose-dependent independent predictor of morbid events; however, other unrecognized confounding variables remain a potential limitation. Of note, our transfusion rate of 63% is much higher than has been previously reported in previous studies [27]. This can be possibly attributed to a number of factors: (1) as a tertiary-care center, some of our patients may have been sicker with a greater comorbidity burden, (2) tranexamic acid use was not approved for use at our institution for the majority of this study, and (3) most studies reporting lower transfusion figures look at intraoperative transfusion while we looked at perioperative transfusion. Additionally, the use of ICD-9 codes to determine morbid events may be less reliable than prospectively collected outcome data. For instance, all of our postoperative infections are grouped such as UTI, surgical site infection and pneumonia with no ability to distinguish them. It is unlikely, however, that such errors would occur in a biased fashion that would influence the primary findings. Another potential limitation of this study is that it represents transfusion practice at one large academic referral center. Therefore, our cohort may not be representative of all individuals at all facilities undergoing spine arthrodesis.

The relationship between transfusion and thrombotic events has direct clinical and economic implications, as average hospital costs are $30,587 greater in patients who develop a DVT after spine fusion than in those who do not [7]. Furthermore, this relationship between transfusion and thrombotic complications may aid clinical decision-making, especially as the use of antifibrinolytic medications becomes more common in spine surgery [5]. The thrombotic complications in our cohort do not appear to be attributed to such medications, for of the 44 patients that received tranexamic acid in our cohort, no thrombotic events occurred. It is possible, however, that we may have underestimated the incidence of thrombotic events if antifibrinolytics had been used with greater frequency, as there are no adequately powered studies to date, proving that this class of drugs does not predispose patients to thrombosis [20].

It has been postulated that transfusion of banked blood may promote thrombosis owing to the increased endothelial adherence and aggregability of RBCs after storage [15]. These effects may become even more relevant because spine surgery patients do not routinely receive preoperative thromboembolic chemoprophylaxis, as the risks of bleeding and neurologic compromise from spinal hematoma are thought to outweigh the benefits of prophylactic anticoagulants. For these reasons, the judicious and timely use of postoperative anticoagulants, as well as other methods such as mechanical compression devices, plays an important role.

Like findings reported previously in the literature, our data show a significant association between perioperative blood transfusion and infectious complications after posterior spinal fusion [16, 25, 32]. Further, the dose-response relationship observed between transfusion volume and infection risk in our cohort is consistent with the findings of Woods et al. [32]. The larger impact identified in their cohort as compared with that in our study may reflect differences in their case and control populations, as the patients with infection had significantly lower preoperative hemoglobin levels and significantly greater intraoperative blood loss than did the controls. In our cohort, transfusion dose was associated with infection, even after risk-adjusting for these and other potentially confounding variables.

A variety of immuno-suppressive mechanisms have been proposed to explain the link between allogeneic transfusion and postoperative infection, including decreased CD4 and interleukin 2 (IL-2) receptor-positive helper cells, increased CD8 suppresser cells, decreased natural killer cells, increased numbers of B cells, decreased IL-2 production, and increased prostaglandin E2 production [2, 6, 17]. Transfusion-related immunomodulation (TRIM) is one of the most widely accepted theories to explain the proinflammatory and immunosuppressive effects associated with transfusion. TRIM describes a “two-insult” model wherein the first insult is the patient’s underlying inflammatory condition from surgery and illness and the second insult is triggered by the transfusion through the above-mentioned effects [29]. Support for the premise that hospital-acquired infection risk decreases with a more restrictive transfusion strategy is most clearly demonstrated in a recent meta-analysis of 18 prospective randomized trials [24]. The analysis showed an odds ratio of 0.82 for all patients and 0.70 for the subgroup of orthopedic surgery patients, supporting the decreased risk of hospital-acquired infection with a restrictive transfusion strategy. This is perhaps the strongest evidence yet to link transfusion and infection risk, as the authors included only randomized trials, thus minimizing confounding by indication, which is common in transfusion studies.

In general, restrictive transfusion practices are supported by multiple randomized trials, [3, 11, 13, 14, 19, 23, 31] but the role of restrictive transfusion during spine surgery specifically has not been determined because this patient population tends to have active and ongoing bleeding [22, 32]. Thus, we cannot strongly support or refute restrictive transfusion thresholds during spine fusion. Perhaps the most relevant prospective trial was that by Carson et al. [3] in a population of elderly hip fracture patients with a high prevalence of cardiovascular disease. They found no improvement in morbidity or mortality, or even in the ability to ambulate after surgery, with a Hb transfusion trigger of 10 g/dL, compared with a trigger of 8 g/dL. However, their study focused on the postoperative rather than the intraoperative period, when patients are less likely to have active bleeding.

To our knowledge, this study represents the largest series to date to examine the association between blood transfusion and noninfectious outcomes after spine fusion. Our findings offer new insights into the risk-benefit balance between anemia, transfusion, and outcomes, and may help inform clinical decision-making. By identifying transfusion as a risk factor for thrombosis and infections, our results may serve to heighten clinician awareness to optimize prevention, diagnosis, and treatment of these complications and thereby improve outcomes after spinal fusion.

## Electronic supplementary material

Below is the link to the electronic supplementary material.

ESM 1 (PDF 1224 kb)

ESM 2 (PDF 1224 kb)

ESM 3 (PDF 1224 kb)

ESM 4 (PDF 1224 kb)

ESM 5 (PDF 1224 kb)

ESM 6 (PDF 1224 kb)
